# Attentive Observation Is Essential for the Misattribution of Agency to Self-Performance

**DOI:** 10.3389/fpsyg.2017.00890

**Published:** 2017-06-02

**Authors:** Shiho Kashihara, Noriaki Kanayama, Makoto Miyatani, Takashi Nakao

**Affiliations:** ^1^Department of Psychology, Graduate School of Education, Hiroshima UniversityHiroshima, Japan; ^2^Institute of Biomedical and Health Sciences, Hiroshima UniversityHiroshima, Japan

**Keywords:** attention, false memory, observation inflation, mirror neuron system, agency, ownership, self-other confusion

## Abstract

Recent studies have repeatedly demonstrated a false memory phenomenon in which people falsely remember having performed an action by oneself when in fact they have only observed the action by another person. We investigated the attentional effect to the action itself on the observation inflation. Fifty-four participants first performed and read actions (Phase 1); then, they observed the action video that showed another’s actions (Phase 2), some of which they had not performed in Phase 1. In the Phase 2, they were required to focus on either the actor’s performance (i.e., attentive observation condition) or irrelevant objects, which were presented in the background (i.e., inattentive observation condition) to modulate their attention. Around 2 weeks later, participants took a surprise source-memory test (Phase 3). In this phase, we asked them to judge whether they “performed,” “read,” or “not presented” the action in Phase 1. Three participants were removed from analysis, because they could not attend Phase 3 within 10–16 days after completion of the second phase. We found observation inflation only in the attentive condition, which contradicted the notions from other false memory studies that showed that attention to the target stimuli reduced false memory in general. We discussed the observation inflation mechanism from the perspective of the “like me” system, including the mirror neuron system, self-ownership, and self-agency.

## Introduction

Recent studies have demonstrated a false memory phenomenon which is thought to be due to self-other confusion in the action memory of healthy individuals (e.g., Lindner et al., 2010, 2016; Schain et al., 2012). This phenomenon has been called the OI, in which people falsely remember having performed an action by oneself when in fact they have only observed the action by another person (Lindner et al., 2010). OI represents that people possibly misattribute the sense of agency of the observed action to the self by just observing other people’s actions. Originally, self-other confusion as an agent of a certain action has been a symptom observed in psychiatric patients, for example, auditory hallucinations in most schizophrenic patients (Nayani and David, 1996). This is caused by a patient’s actual utterances, as stated by previous researchers (e.g., McGuigan, 1966; Green and Preston, 1981). Thus, “self-other confusion” as one of a symptom means the confusion of agency judgment “who is the agent of a certain action.” Healthy adults do not likely to confuse own action with others at the moment, however, such confusion can occur in memory.

Since the first study addressing this phenomenon, which developed and used methodology to approach it (i.e., Lindner et al., 2010), OI has been demonstrated per the following experimental paradigm: first, participants perform or read simple action statements (Phase 1; e.g., “shake the bottle!”). Then, they are asked to observe video clips that show another person’s actions (Phase 2). Two weeks later, they take a surprise source-memory test where they are asked to judge whether they “performed,” “read,” or “not presented” the action in Phase 1 (Phase 3). OI is thought to arise when they believe they performed some of the actions in Phase 1 that in fact they only observed in Phase 2.

Previous researchers studying OI have demonstrated that both facilitating and disturbing factors affect this misattribution during observation of another’s actions. When we observe another’s action, we can obtain information to induce a feeling “as if I do it,” whereas we can find any clue to be conscious of the fact that the agent of the action is other. These ideas have already been advocated in the “like me” hypothesis, which is a system to determine whether a certain agent is close to oneself (Meltzoff, 2007).

Regarding OI’s facilitating factor, it has been suggested that motor simulation using the MNS, which is activated both during performing an action and observing another’s action (Rizzolatti and Craighero, 2004), is one of the critical processes that induce false memories of self-performance (e.g., Lindner et al., 2016). Much evidence has shown the overlap of neural activation during the performance of an action and during the observation of another’s action (e.g., Grèzes and Decety, 2001); therefore, it has been considered that motor representation is created during one’s own action performance and likewise during observation of another’s actions. Previous studies on OI suggested that motor representation created by motor simulation induces the false attribution of self-performance (Lindner et al., 2016).

Regarding OI’s disturbing factor, previous research showed that the information in the action video indicating “the actor is not me” decreases the occurrence rate of this misattribution. For example, Lindner et al. (2012) manipulated group membership by actor’s complexion (dark vs. fair), and found that when fair-skinned participants observed actions performed by a dark-skinned actor (i.e., out-group actor for participants), the rate of OI was significantly decreased. In addition, Schain et al. (2012) suggested that when the action video showed an actor’s face (vs. concealing the actor’s face), the rate of OI was significantly reduced. Previous research on a sense of ownership has suggested that body ownership illusion on virtual objects decreased when the object was a black cuboid (“it is not like my own body”) compared with when the object was a dummy body (“it is like my own body,” Lenggenhager et al., 2007).

Given that OI could be induced during observation of “another’s action,” the observed body is not, in principle, the observer’s body; however, it contains many characteristics indicating the fact that “it is not the observer’s body.” While attentive observation of only the target action itself may be likely to increase OI because of facilitation of MNS, careful observation of actor in OI paradigm (Lindner et al., 2010) will provide participants with not only motor information but also information about actor’s visual features. If so, it is possible that the careful and attentive observation of the other’s action decreases the occurrence of OI because participants can feel less ownership of the people in action video. Schain et al. (2012) examined the effect of the actor’s face on OI manipulating attentional focus to the action video. They used three experimental conditions. In the first (the face-invisible condition), the actor’s face could not be observed by participants. In the second (the face-visible/action-focus condition), the actor’s face could be observed by the participants and they were asked to focus on the actor’s action. In the third (the face-visible/face-focus condition), the actor’s face could be observed by the participants and they were asked to focus on the actor’s face. Consequently, in the face-visible/face-focus condition, the occurrence of OI was eliminated. In addition, even if participants focused on the action, the appearance of another’s face in the action video (i.e., in face-visible/action-focus condition) decreased OI occurrence rate compared to the face-invisible condition. Per these results, they concluded that attention on the other’s face is a crucial factor to disturb OI.

However, Schain et al.’s (2012) experimental design had a possibility to confound two types of attentional effects: the first was attention on the actor’s face as to disturb illusory ownership on an actor in the action video (face-visible/face-focus > face-visible/action-focus > face-invisible condition); the second, was attention on the action itself as a factor to facilitate false agency attribution on the other’s action (face-invisible ≥ face-visible/action-focus > face-visible/face-focus condition). That is, it still is not clear how attention to the action itself affects the occurrence of OI. Schain et al.’s (2012) findings may be due to the use of a unique stimulus of the face as a distractor. In accordance with Leonetti et al. (2015), MNS activation is enhanced by peripheral vision. In other words, OI should occur in a situation where the observer’s attention is not directed to an action itself (i.e., the inattentive observation condition).

In this study, we modulated observer’s attention and investigated the impact of the attention on OI to elucidate the top-down influence on the agency misattribution without any modification of the video contents. We focused on the effect of attention on the action itself using visual distractor unrelated to the actor in the action video, instead of the actor’s face. We instructed participants to focus on the objects appearing in the background of the action video to investigate the attentional effects of other’s actions on OI.

## Materials and Methods

### Participants

Fifty-four healthy undergraduates (29 females, age range = 18–22 years, mean age = 20.3 years, *SD* = 1.2) participated in our experiment. This study was conducted per the recommendations of the Research Ethics Committee of Hiroshima University with written informed consent from all participants. This study was conducted in accordance with the Declaration of Helsinki.

### Design

We used a one-way design (observation condition: attentive observation vs. inattentive observation) manipulated within-participants. Both observation conditions used a randomized block design. In the attentive observation condition, participants were instructed to focus on the action of an actor in the video while ignoring objects appearing in the background that were unrelated to the task and actor. In the inattentive observation condition, participants were instructed to focus on some objects in the background of the action video.

### Materials

**FIGURE 1:**
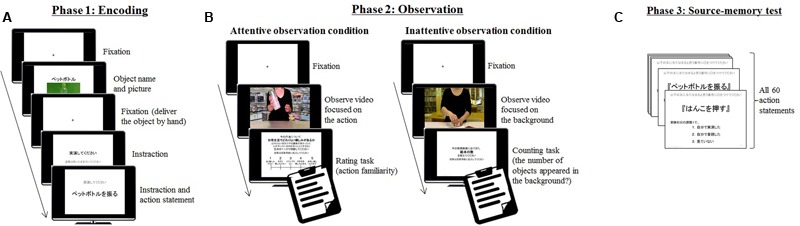
The flow of the procedure. **(A)** Demonstrates the flow of Phase 1, where participants performed or read action statements. **(B)** Demonstrates the flow of Phase 2, including the observation of other’s action. **(C)** Demonstrates the flow of Phase 3, where participants completed a source-memory test including whether they performed, read, or did not see a certain action statement in Phase 1. This phase was conducted 2 weeks after Phases 1 and 2.

We generated 60 action statements and action videos consistent with Lindner et al. (2010). The action statements described actions to manipulate objects (e.g., “shake the bottle” in Japanese). Each action video was the 15-s composite video that randomly combined 60 movies showing the actor’s action performance with 30 landscape photographs by using Adobe After Effects CC 2014.1.1 (13.1.1, Adobe Systems Software Ireland Ltd.: see **Figure 1B**). To distract participants’ attention from the actor’s action, 6–10 unrelated objects per video randomly appeared in the background of the video (e.g., some books appeared in the picture of the library as part of the background).

The action video was made in accordance with Lindner et al. (2010): that is, the video filmed a female actor’s torso, arms, and hands from a third-person perspective. In each video, she performed the actions described in the action statements. Importantly, to conceal the actor’s facial characteristics, we omitted the actor’s face from the action video. To strengthen homogeneity of the materials, only one female actor performed all actions in the video.

### Procedure

The experiment was controlled by a computer and consisted of three phases following Lindner et al.’s (2010) experimental paradigm (**Figure 1**). In Phase 1, in accordance with the previous research, we set the condition for asking participants to read aloud action statements (read condition) in addition to the condition for actually performing an action themselves (perform condition) to secure the task-difficulty. In Phase 2, two observation condition (attentive vs. inattentive) were prepared to investigate the attentional effect. In Phase 3, which was conducted 2 weeks later by Phase 2, we measured their memory for self-performance in Phase 1. The action statements were counterbalanced across participants.

**Figure 1A** shows the flow of Phase 1. In the first phase, participants performed 15 actions, and read 15 action statements aloud. The item lists shown in each encoding condition were randomly chosen from all 60 action statements, and they were presented at the center of a 24″ BenQ LCD Monitor display in a random order. We presented the following stimuli using Microsoft PowerPoint 2010, which was manually operated by the experimenter. At the beginning of each trial, the experimenter handed an object (e.g., a plastic bottle filled with water) directly to participants after its name and picture appeared on the screen. Then, Japanese instruction to the next action statement appeared on the screen. The instruction meant either “please perform” or “please read.” After that, the experimenter told them to obey the instruction (i.e., perform or read the action statement) for 15 s [during this time, the monitor showed both the instruction and the action statement (e.g., “shake the bottle” in Japanese)].

Between the first and second phase, a 5-min arithmetic task was administered as a distractor. **Figure 1B** indicates the flow of Phase 2. In the second phase, participants observed the 15 action videos per condition that showed other’s actions. Some videos presented in this phase were not performed earlier (i.e., 5 action statements were performed, 5 were read, and 5 were not performed in Phase 1). In this phase, participants were required to pay attention to an actor’s performance (attentive observation condition) or the irrelevant objects, which were presented in the background (inattentive observation condition) while watching the action video. Each observation condition had a different task after watching the video: participants rated the familiarity with the action in everyday life on a five-point Likert scale (attentive observation condition), or participants reported the number of objects that appeared in the background in the video.

**Figure 1C** demonstrates the flow of Phase 3. The third phase was conducted 10–16 days after the first and second phases. Participants were invited to the laboratory to participate in another experiment and took a surprise source-memory test for all 60 action statements. At the source-memory test, they were asked to judge whether they performed or did not perform (read or not presented) each action described in the statement presented in Phase 1.

According to Lindner et al. (2010), the occurrence rate of OI was calculated as follows: (a) all action statements were assigned into two categories [actually performed/not performed (i.e., read or not presented) in Phase 1], (b) the performed-response (i.e., participants labeled as “I have done the action in Phase 1” in Phase 3; **Table 1**) to the action statements that were not performed in Phase 1 was considered as a false-response, and (c) the subtraction of the proportion of the false-response not observed in Phase 2 from the proportion of the false-response observed in Phase 2 was defined as the OI effect.

**Table 1:** Mean proportion of performed-responses as a function of Phase 1 encoding and Phase 2 observing.

Phase 1: encoding	Phase 2: observing		
	Attentive	Inattentive	No observation
Performed	0.91 (0.12)	0.84 (0.17)	0.82 (0.20)
Read	0.14 (0.15)	0.04 (0.08)	0.04 (0.09)
Not presented	0.04 (0.10)	0.02 (0.05)	0.00 (0.01)

*Performed-responses means the responses that participants labeled as “I have done the action in Phase 1” in Phase 3. All variables varied within participants. Standard deviations are given in parentheses.*

We analyzed participants’ OI rate in each observation condition. To investigate the differences in OI between both observation conditions, we conducted paired *t*-tests. In addition, we conducted a one-sample *t*-test to confirm the occurrence of OI in each observation condition. The alpha level was set at α = 0.05. All analyzes were conducted by R studio (R Core Team, 2016). Furthermore, we adopted Cohen’s *d* as an effect size of *t*-tests calculated by the R package “compute.es” (Del Re, 2013).

## Results

All participants who joined the first and second phase of the experiment took part in the third phase. Three participants could not attend the third phase within 10–16 days after from the completion of the second phase. Therefore, we conducted the analysis for the data from 51 participants.

**Table 1** shows the mean proportions of participants’ performed-responses in Phase 3.

**FIGURE 2:**
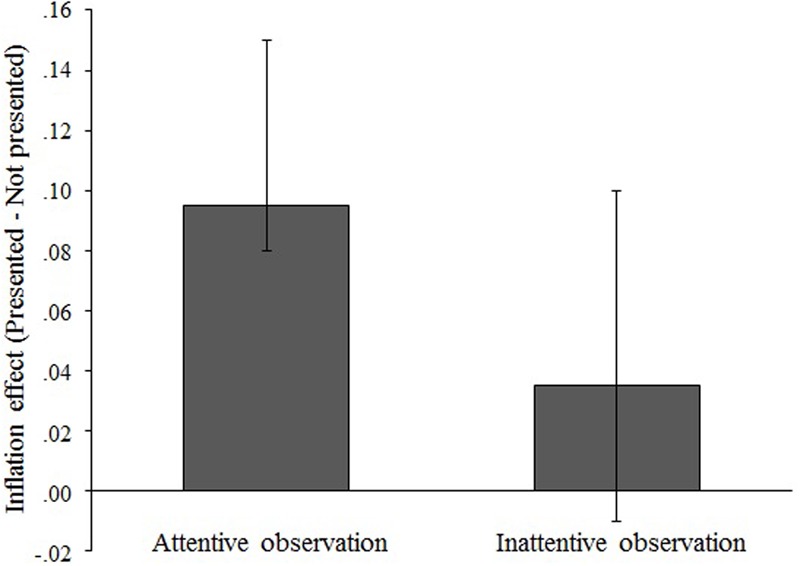
The magnitude of the inflation effect. Results are shown for both Phase 2 observing conditions. Each value shows the (pseudo-) median calculated by the Wilcoxon signed-rank test. The magnitude of the inflation effect was calculated as the difference between the proportion of false performed-responses for action statements presented in Phase 2 and the proportion of false performed-responses for corresponding action statements not presented in Phase 2. Error bars represent the Wilcoxon signed-rank 95% confidence intervals.

The OI effect in each observation condition demonstrated in **Figure 2**.

The Shapiro–Wilk test revealed that the data did not satisfy the assumption of a normal population (for the size of the OI effect in the attentive condition, *w* = 0.90, *p* < 0.001; in the inattentive condition, *w* = 0.75, *p* < 0.001); therefore, we applied the logarithmic transformation.

We found that OI was significantly larger in the attentive condition than it was in the inattentive condition [*t*(50) = 5.35, *p* < 0.001, *d* = 1.06]. In addition, we conducted one-sample *t*-tests to ascertain whether OI significantly occurred in each condition. Thereby OI was found in the attentive condition [*t*(50) = 5.70, *p* < 0.001, *d* = 1.13], but not in the inattentive condition [*t*(50) = 1.93, *p* = 0.06, *d* = 0.38].

Since the data did not satisfy the assumption of a normal population, we also conducted a non-parametric test (i.e., Wilcoxon signed-rank test) on the OI occurrence rate just to be certain. We also found a trend similar to the results of parametric tests in non-parametric tests: There was still significant differences in the occurrence of OI for the difference between the two observation conditions (*V* = 383, *p* < 0.001), for a one-sample test in the attentive condition (*V* = 672, *p* < 0.001). Note that there was also a significant difference in a one-sample Wilcoxon signed-rank test in the inattentive condition (*V* = 145, *p* = 0.04). However, this result contains 0 in the 95% confidence interval [95% CI = (-0.01, 0.10)].

## Discussion

The aim of this study was to confirm the pure effect of attention to other’s action on OI, expanding the findings in Schain et al. (2012).

Observation inflation effect occurred at a significantly higher rate after attentive observation of another’s action video compared with after inattentive observation. Considering the studies on MNS, which demonstrated the enhancement of MNS activations when participants observed target action attentively (Muthukumaraswamy and Singh, 2008), attention to action could facilitate “like me” judgment even if it was a misattribution; therefore, greater OI might arise. However, it cannot be said that the MNS activity declined in the inattentive observation condition per the evidence on motor resonance indicating that peripheral vision facilitates the activation of motor resonance, which are supported by MNS activity (Leonetti et al., 2015). Given the circumstances, conversely, another possibility is considered: higher-order cognitive control. Brass and Haggard (2008) and Brass et al. (2009) have suggested that the higher brain mechanism that judges agency (e.g., TPJ) also monitors or controls the agency judgment supported by lower-order system for motor simulation (e.g., MNS). Considering this, the agency judgment confusion occurred at a different cognitive-hierarchical level, which would involve TPJ regardless of MNS activation in the inattentive observation condition.

Further findings from a one-sample *t*-test showed that significant OI was found only in the attentive observation condition. This finding indicates that directing the attention to the action performed by others is a requisite condition for OI occurrence. Typical memory studies have demonstrated that attention to the target content could help us to keep the content in mind (e.g., Craik et al., 1996; Berryhill et al., 2011). Although it seems inconsistent with the typical theory of memory function, our results have higher affinity with the findings about sense of ownership or agency (e.g., Haggard and Cole, 2007; Lenggenhager et al., 2007; Moore and Fletcher, 2012; Kokkinara et al., 2016). In previous research on ownership or agency, it has been suggested that directing attention to a target was related to the occurrence of a sense of agency or ownership. For example, Haggard and Cole (2007) used the method called “intentional binding effect,” which was one of the objective ways to investigate participants’ sense of agency, and showed that the binding effect was increased when participants could focus their attention to stimuli. Furthermore, in previous studies on memory of involuntary actions, it was suggested that a voluntary action that attends to a sense of agency affects the memory of involuntary actions, which never have sense of agency (e.g., Jensen et al., 2014; Khalighinejad and Haggard, 2016). Altogether, this false attribution of self-performance may not just be typical false memory, but also something concerning the sense of ownership as a distracting factor or agency when observing another’s action as a facilitating factor.

From this perspective, as discussed in the introduction section, OI could be decreased by a disrupted sense of ownership, which was induced by an actor’s face (Schain et al., 2012) or skin color as a clear indicator of “not like me.” Whereas, our results suggested that the decreased OI from focusing on the actor’s face in Schain et al. (2012) can be explained by just distracting participant’s attention from the action itself. It is indicated that the effect of attention to OI will be determined by the amount of the attention to the action itself rather than by the amount of attention is paid to the visual appearance that allows us to discriminate self from other.

As discussed above, we have suggested new insights concerning the OI mechanism. First, it was not a typical false memory because the occurrence rate was high when participants paid attention to the target. Second, it could be worthy to reconsider the OI mechanism from the perspective of the “like me” system, including MNS, self-ownership, and self-agency. Per this perspective, we propose the process of the occurrence of OI in the OI paradigm (Lindner et al., 2010) as follows: (1) First, participants get a sense of agency to their own action when they performed some actions in Phase 1. (2) Then, they can have a vicarious-agency (Wegner et al., 2004) to observed other’s actions by the motor simulation based on MNS when they directed their attention to target action during observing the other’s action in Phase 2. (3) The judgment of “who is the agent,” that is, “the agent is me or not me” is started in conjunction with the second process. If they recognize the obvious “sense of others” at this point, such as the actor’s face, clothes or complexion, the vicarious sense of ownership to an actor’s body can be remarkably disturbed. (4) Finally, the misattribution for self-performance on the action that they did not perform, namely, the OI arises at the source-memory test in Phase 3 when they confuse a real agency gained in Phase 1 and vicarious agency accidentally obtained in Phase 2 during remembering their action in Phase 1. It is possible that the OI never occurred when they made the judgment of “the agent was other” (i.e., they inhibited their ownership to observing another) in the third process.

## Conclusion

We shed light on the possible relationship between self-ownership/agency and false agency attribution in memory, namely OI, and investigated the pure effect of the attention to the action itself. We demonstrated the effect of attention to the action itself as a fundamental factor to induce OI. Given that attentive observation of another’s action could facilitate MNS activation as a lure to misattribute the other’s action to our self, our findings might reflect that MNS activation facilitates the occurrence of OI. On the other hand, it is possible to form a different interpretation. Given that motor resonance is thought to reflect MNS activation to facilitate in peripheral vision (Leonetti et al., 2015), and that there may be a higher cognitive mechanism for self-other distinction controlling our self-agency judgment (Brass et al., 2009), our result might be explained by another mechanism [i.e., the agency-judgment mechanism including TPJ suggested by Brass et al. (2009)] even though MNS is actually activated in the inattentive condition. However, our study did not directly modulate and measure MNS, so it cannot be mentioned properly. Furthermore, it is conceivable that the instruction in the experimental procedure may affects the occurrence of OI. In future, it is necessary to carry out OI experiments with full attention to the influence of the instruction, such as translation. Further investigations are required to directly examine the relationship between OI and MNS as an index of agency misattribution with participants’ self-reports (e.g., Tieri et al., 2015) or other indirect methods (e.g., intentional binding; Haggard et al., 2002; Haggard and Cole, 2007). Then, we can better understand the mechanisms of agency misattribution.

## Author Contributions

SK designed the experiment, collected and analyzed the data, and wrote the manuscript. NK, MM, and TN reviewed and revised the manuscript. All authors approved the manuscript.

## Conflict of Interest Statement

The authors declare that the research was conducted in the absence of any commercial or financial relationships that could be construed as a potential conflict of interest.

## Abbreviations

OI: observation inflation effect
MNS: mirror neuron system
TPJ: temporo-parietal junction
